# Obstacles to Brain Tumor Therapy: Key ABC Transporters

**DOI:** 10.3390/ijms18122544

**Published:** 2017-11-27

**Authors:** Juwina Wijaya, Yu Fukuda, John D. Schuetz

**Affiliations:** Department of Pharmaceutical Sciences, St. Jude Children’s Research Hospital, 262 Danny Thomas Place, Memphis, TN 38105-2794, USA; juwina.wijaya@stjude.org (J.W.); yu.fukuda@stjude.org (Y.F.)

**Keywords:** ABC transporter, CNS, blood–brain barrier, blood–tumor barrier, glioma, medulloblastoma, chemotherapy

## Abstract

The delivery of cancer chemotherapy to treat brain tumors remains a challenge, in part, because of the inherent biological barrier, the blood–brain barrier. While its presence and role as a protector of the normal brain parenchyma has been acknowledged for decades, it is only recently that the important transporter components, expressed in the tightly knit capillary endothelial cells, have been deciphered. These transporters are ATP-binding cassette (ABC) transporters and, so far, the major clinically important ones that functionally contribute to the blood–brain barrier are ABCG2 and ABCB1. A further limitation to cancer therapy of brain tumors or brain metastases is the blood–tumor barrier, where tumors erect a barrier of transporters that further impede drug entry. The expression and regulation of these two transporters at these barriers, as well as tumor derived alteration in expression and/or mutation, are likely obstacles to effective therapy.

## 1. ATP-Binding Cassette (ABC) Transporters in the Central Nervous System (CNS)

### 1.1. Overview of Blood–Brain Barrier (BBB)

**Figure 1:**
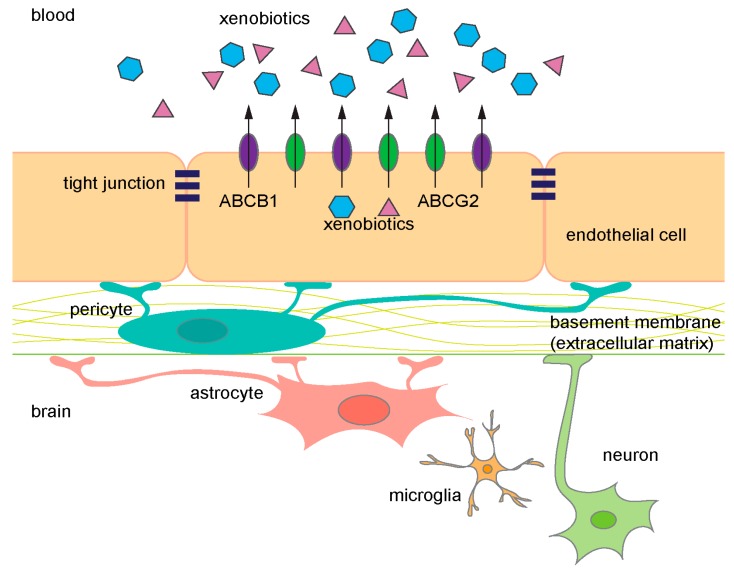
Diagram of the components in the blood–brain barrier (BBB). ABCB1 (purple) and ABCG2 (green) are expressed on the luminal side of endothelial cells. They prevent xenobiotics (blue and pink) from entering the brain and frequently share common substrates. ABC, ATP-binding cassette.

The central nervous system (CNS) is separated from the blood by a vasculature comprised of highly specialized endothelial cells (ECs) that form part of the blood–brain barrier (BBB) (Figure 1). The BBB provides nutrients to the CNS by selective uptake of small molecules but also protects the CNS from deleterious endogenous and xenobiotic molecules. Importantly, transporters protect the CNS, by actively extruding xenobiotics at the BBB into the blood to create a “pharmacological sanctuary” for the brain. However, this sanctuary for the brain creates an obstacle to the effective delivery of drugs necessary for treating pathological conditions of the CNS from neurological diseases to cancer. 

The BBB was functionally identified in the early 1900s when an intravenous injection of various dyes failed to stain the CNS, whereas the dyes injected intrathecally into the subarachnoid space stained the brain [1]. In 1934, Stern proposed that the BBB not only protected the brain from toxic compounds, but also to maintaining homeostasis of the brain (as cited in [1]). 

About 5% of the brain is composed of vascular cells and these cells provide an almost seamless protective barrier, the BBB [2,3]. Specialized ECs line the cerebral blood vessels, and properties of these cells, such as the expression of ATP-binding cassette (ABC) transporters, facilitate extrusion of undesirable molecules providing one level of barrier protection. A second level of protection is provided by a property of the ECs. Unlike ECs in other peripheral vessels, the cerebral vessel ECs form intercellular tight junctions (TJ) that are continuous. In combination, transporters and TJs provide a dual barrier to limit compounds from accessing the brain parenchyma. The integrity and maintenance of the BBB is further supported by astrocytes, neurons, microglial cells, pericytes, and the extracellular matrix (Figure 1) [4]. 

#### 1.1.1. Development and Regulation of the BBB

The BBB develops by a multistep process requiring interplay among metabolic and signaling pathways emanating from a variety of cell types. Angiogenic stimuli from neural progenitor cells produce new vessels, sprouting from pre-existing vessels, equipped with properties of the BBB. The barrier function and integrity mature to provide maximum effect and must be maintained through extra- and intra-cellular signaling. Interestingly, some of these processes are disrupted in CNS tumors, as described later.

In brain ECs, the Wnt-β-catenin pathway promotes angiogenesis during embryogenesis [5]. Indeed, the transcriptional profile of brain ECs is enriched in the Wnt-β-catenin signaling pathway compared to peripheral ECs [3]. In the canonical Wnt pathway, secreted Wnt ligands bind to the Frizzled receptor on ECs plasma membrane. Binding of Wnt to the Frizzled receptor blocks phosphorylation of β-catenin by glycogen synthase kinase 3 (GSK3), allowing β-catenin to accumulate and translocate to the nucleus where it binds to TCF/LEF (T-cell factor/lymphoid enhancer factor) transcription factors to modulate expression of target genes [6]. The Wnt-β-catenin pathway also enhances BBB function by upregulating the expression of BBB-specific genes such as *Glut-1* (*Slc2a1*), a nutrient uptake carrier. In addition, claudin-3 expression is increased by β-catenin. The Wnt-β-catenin pathway is implicated in the regulation of TJ formation during BBB maturation, because loss of β-catenin at postnatal days 4, 7, and 14, corresponding to days of BBB maturation, results in decreased claudin 3, an essential component of the BBB TJ [7]. This reduction in claudin 3 level associates with a decrease in BBB integrity as measured by evans blue staining [7]. Taken together, the Wnt-β-catenin pathway promotes angiogenesis and TJ formation at the BBB.

The sonic hedgehog (SHH) pathway also regulates BBB development. Unlike the Wnt-β-catenin pathway, SHH signaling is not required for angiogenesis in the brain, but it regulates the TJ function through the expression of TJ proteins such as occludin [8]. SHH secreted from astrocytes binds and inactivates the SHH receptor patched 1 (PTCH1) on the cell surface of brain ECs. PTCH1 then releases the co-receptor smoothened (SMO) to activate a downstream signaling cascade leading to the translocation of the transcription factor GLI (glioma-associated oncogene)into the nucleus. Blocking SHH signal transduction, by cyclopamine, an inhibitor of SMO, produces an extravasation of exogenous and endogenous molecules secondary to breakdown of the BBB. As brain hemorrhage was not observed, these results suggest SHH signaling ensures the integrity of brain EC tight junctions. 

The EC transcriptome of BBB differs from peripheral ECs. The EC transcriptome displays a regulatory signature: the nuclear receptor, retinoic x receptor (RXR)/pregnane x receptor (PXR) and the “LPS (lipopolysaccharide)/IL-1 (interleukin-1) mediated inhibition of RXR function” pathway when compared to the peripheral ECs. The nuclear receptor RXRα regulates expression of brain EC-enriched transcripts, including transporters *Abcb1* and *Abcc4* [3]. Notably, while the RXRα-mediated transcription is upregulated in brain ECs, peripheral ECs downregulate these pathways. 

Pericytes and astrocytes contribute to BBB development and maintenance. Pericytes envelope the outer walls of capillaries and provide structural stability. Pericytes have more than a physical role and are required for proper BBB development during embryogenesis [9]. Pericytes affect vessel permeability by regulating TJ formation. Astrocytes, on the other hand, are absent during initial vascularization, but maintain BBB function and integrity once formed. Astrocytes, as mentioned above, secrete signaling molecules such as SHH that support TJ integrity.

Exactly how the BBB protects the developing brain has been an incremental process. Wislocki, in 1920, showed that the brain from a mid-gestation guinea embryo was refractory to dye staining, thus revealing, by intravenous trypan blue (a molecule < 1000 Da) the BBB during fetal development (as cited in [1]). Similar results were obtained in human fetuses, demonstrating the conservation of BBB function in mammals. Ultrastructural studies revealed that TJ are formed in rat and rabbit embryos suggesting that this component of the BBB forms early in development [1,10]. Interestingly, the expression of genes for uptake and efflux transporters, in ECs vary during development into adulthood, possibly contributing to the notion of “immaturity” of the fetal or post-natal BBB compared to the adult BBB. As expected, some uptake carriers that provide nutrients are higher in fetal brain ECs. These are likely important in increasing the brain concentration of certain amino acids, but suggest the BBB is “leaky”. An efflux transporter *Abcb1a* transcript increases in adult mice compared to post-natal pups, which is likely to selectivity restrict the molecules allowed into the brain. 

#### 1.1.2. Disruption of the BBB in Diseases

The BBB integrity and functionality must be maintained throughout life to protect CNS against a host of toxins and pathogens; however, some insults and diseases compromise the BBB. Oxidative stress is known to disrupt BBB and is commonly seen in neurological disorders such as multiple sclerosis and stroke [11,12]. Reactive oxygen species (ROS) generated during oxidative stress can also disrupt the BBB [13]. Interestingly, increased ROS accumulation is associated with aging, thus it is conceivable that the BBB integrity and functionality declines as people age (discussed further in Section 1.3). The CNS tumors such as glioblastoma and Wnt-subtype of medulloblastoma are also known to disrupt BBB function via different mechanisms that will be described later (Section 2 and Section 3).

### 1.2. ABC Transporters Provide Critical Barrier Function of BBB

Efflux transporters that belong to the ABC transporter superfamily provide critical barrier function at the brain ECs through the efflux of toxic compounds. In particular, two transporters, ABCB1 (also known as P-glycoprotein or multidrug resistance protein 1 (MDR1)) and ABCG2 (breast cancer resistance protein (BCRP)) are highly expressed in the brain ECs. Other ABC transporters such as ABCC2, ABCC4, and ABCC5 have been detected, but this review will primarily focus on ABCB1 and ABCG2, as they are also the major transporters involved in multidrug resistance (MDR) due to their ability to transport a wide range of chemotherapeutics. Transcriptomic and mass-spectroscopy analysis showed enrichment of both *Abcb1* and *Abcg2* mRNA and protein in the brain vessels further confirming the importance of these transporters in BBB function. Of interest, ABCC4 is highly expressed in the choroid plexus and plays a critical role in providing barrier function at the blood–cerebrospinal fluid (CSF) barrier. At the BBB, ABCC4 protein levels were >10-fold lower than ABCB1 and ABCG2 [14], which may be an indication for its specific role in blood–CSF barrier. A discussion of ABCC4 is outside of the scope of this review. In the following sections, we will highlight the expression, localization, and clinical relevance of these transporters at the BBB. 

#### 1.2.1. ABCB1 Expression at the BBB

ABCB1 was the first member of the ABC protein family to be identified at the BBB. ABCB1 was first implicated at the BBB by immunocytochemical and immunohistochemical studies of ABCB1 in human and rat brain tissues [15,16]. Notably antibodies against ABCB1 only detected it at the brain capillaries but not capillaries of non-CNS tissues. In mouse and rat, there are two ABCB1 isoforms, encoded by *Abcb1a* and *Abcb1b* genes with *Abcb1a* being the predominant gene expressed at the BBB. Mouse ABCB1A’s amino acid sequence and gene regulation parallels human ABCB1. In mouse, the *Abcb1a* transcript was one of the most enriched transcripts in brain ECs compared to pericytes, and its selective expression in brain ECs compared to liver and lung ECs makes it an excellent BBB marker. In situ hybridization revealed that *Abcb1a* expression is detected as early as E10.5 of mouse embryos in the neural tube [17]. In human brain microcapillaries, ABCB1 protein was detected by mid-gestation using immunohistochemistry. Adults might have greater ABCB1 amounts. For example, a quantitative Western blot analysis showed that ABCB1 protein amount in the BBB of adult rats was about five-fold higher than that of the 9- and 17-day-old rats [18,19]. Accordingly, mouse *Abcb1a* mRNA expression showed an increased expression in adult animals compared to post-natal pups [3]. Finally, ABCB1 function in adult mouse was greater than post-natal animals as determined by administration of an ABCB1 inhibitor, cyclosporine A coupled with the ABCB1 substrate, digoxin. Specifically, the brain to blood/plasma ratio, at 2 h post-administration, were 80–90% lower in the adult brain compared to the postnatal day 1 (P1) brain [20]. *Abcb1a*-deficient mice showed no difference, regardless of the age indicating that the increased transport activity, measured by digoxin, was entirely due to ABCB1 and not another transporter in adult mice.

#### 1.2.2. Cellular Localization of ABCB1

Initially, ABCB1’s function at the BBB was only speculative because the immunohistochemical analysis had a limited ability to establish ABCB1 subcellular localization in the brain EC. This problem was resolved by immunoelectron microscopy with a monoclonal antibody against ABCB1 that localized it to the luminal membrane of human brain capillaries. Confocal immunofluorescence microscopy using a different monoclonal antibody also selectively stained ABCB1 at the luminal side of the brain ECs in rat, lending further support for its putative role in providing a transport barrier function at the BBB. Further, Beaulieu and colleagues fractionated brain capillaries in an effort to physically localize ABCB1. The luminal membrane from brain capillaries was enriched with ABCB1 [21]. To achieve enrichment, the endothelial luminal membrane was coated with a cationic colloidal silica, allowing selective isolation of the luminal membrane due to a difference in the density. A known capillary luminal membrane protein, glucose transporter 1 (GLUT1), was also enriched in the luminal membrane fraction, thus confirming the purity of the isolation. These studies provided strong evidence that ABCB1 is highly expressed at the BBB on the luminal side, suggesting it pumps toxic compounds back into the blood.

#### 1.2.3. ABCB1 Function at the BBB and Clinical Importance

The *Abcb1a*-deficient mice provided definitive evidence that the function of ABCB1 at the BBB was to restrict drug penetration into the brain. Under normal housing conditions, the *Abcb1a*-deficient mice were phenotypically normal, in both appearance and behavior, until an inadvertent exposure to ivermectin, an agent that is routinely used to eradicate mites, proved lethal. With ABCB1, ivermectin is not a neurotoxin, but in its absence, ivermectin brain levels climb to nearly 100-fold higher in the *Abcb1a*-deficient mice compared to wild-type mice [22]. Subsequent study showed that ivermectin is transported by both mouse and human ABCB1. Since the serendipitous discovery of ABCB1 critical role in protecting the brain from ivermectin, many more drugs have been shown to be extruded from the brain by ABCB1. Incidentally, an inbred strain of CF1 (Carworth Farms) mice harbor an *Abcb1a* mutation that renders ABCB1 non-functional [23,24] and a Collie subpopulation has a non-functional ABCB1 due to a −4 bp deletion producing a frameshift and a premature stop codon [25]. These animals were also hypersensitive to ivermectin, indicating functional conservation of ABCB1. Ivermectin has been used to treat humans for a worm infection, referred to as African river blindness; however, it has not been determined if humans that are highly sensitive to this treatment have loss of ABCB1 function. The impact of loss of ABCB1 function in human BBB has not been extensively studied. The exome variant server (http://evs.gs.washington.edu/, accessed on 8 August 2017) shows over 70 single nucleotide polymorphisms (SNPs) in *ABCB1* that result in missense mutations. It is conceivable that some of these SNPs result in non-functional ABCB1. Perhaps future studies will use this SNP data as a starting point to prospectively identify individuals with a defective ABCB1 that might be susceptible to drug-induced CNS toxicities.

#### 1.2.4. ABCG2 Expression at the BBB

ABCG2, which was first named the breast cancer resistance protein (BCRP) for its high expression in an anthracycline resistant breast cancer cell line, is an ABC transporter expressed at the BBB. In human brain, ABCG2 transcript and protein was detected [26], and, importantly, immunohistochemistry showed it primarily localized to the luminal side of brain capillaries. This places ABCG2 in the same location as ABCB1 in the BBB, suggesting a similar role in exporting compounds toxic to the CNS.

*ABCG2* expression in the developing brain EC of humans is detected early (mid-gestation-22 weeks), and in mid-gestation for mouse and rat (E12.5), but the amount does not appear to increase during development, unlike ABCB1. Although, *Abcg2* transcripts increase in brain ECs from adult mice compared to post-natal pups the changes are non-significant [3]. Furthermore, functional assessment of ABCG2 revealed no difference in ABCG2-mediated prazocin transport between adult rats and P21 pups [27]. These results suggest, unlike ABCB1, that there is no developmental upregulation of ABCG2 and that it maintains peak function even early during development.

#### 1.2.5. ABCG2 Function at the BBB; Redundancy with ABCB1?

ABCB1 and ABCG2 might have redundant roles at the BBB, as suggested by, brain capillaries from *Abcb1a*-deficient mice expressing ~3-fold higher *Abcg2* transcripts compared to wild-type mouse brain capillaries [28]. We suggest that such compensation by ABCG2 provides protection of the fetal brain before ABCB1 expression reaches its peak in adulthood. Recently, positron emission tomography (PET) and radiotracers for tariquidar and elacridar revealed functional interplay between ABCB1 and ABCG2 at the human BBB [29]. The CNS distribution of these ABCB1 and ABCG2 inhibitors was assessed in healthy volunteers that differed in ABCG2 function (one group harbored an allele (*c.*421 C > A) that is functional impaired, the other group was *ABCG2* wildtype). Administration of an ABCB1 inhibitor alone did not change the brain distribution of the radiotracers, whereas brain accumulation in the volunteers harboring the *c*.421 SNP was greater than the volunteers who are wild type for *ABCG2*. These results demonstrated that for some drugs ABCB1 and ABCG2 are functionally redundant in humans. Moreover, these results demonstrated that inhibition of both transporters is required to achieve effective CNS levels when drugs are substrates for both transporters. Furthermore, these results revealed the potential risk for neurotoxicity among individuals with a non-functional *ABCG2* allele. 

### 1.3. Regulation of ABC Transporters at the BBB

Ligand-activated receptors and inflammatory pathways are among those pathways that can elicit a rapid change in expression of drug-efflux transporters at the BBB. Additionally, studies have also suggested that gender affects expression and activity of both ABCB1 and ABCG2. Understanding how these transporters are regulated will provide insight into additional strategies capable of altering BBB integrity and improve the delivery of drugs to the brain. 

#### 1.3.1. Ligand-Activated Receptor Signaling

The nuclear receptors are a group of ligand-activated receptors that are activated by endogenous ligands (e.g., hormones and steroids) and xenobiotics [30]. In some cases, ligand binding to the receptor induces a conformational change that initiates receptor translocation to the nucleus where it forms a complex with various co-activators or co-repressors to either induce or repress transcription of target genes [30]. A subset of these nuclear receptors is expressed at the BBB and can regulate ABCB1 and ABCG2 levels.

PXR, arylhydrocarbon receptor (AhR), and constitutive androstene receptor (CAR) can affect both human and mouse ABCB1 expression at the BBB. The antibiotic rifampicin is a potent ligand for human PXR, which binds DNA elements referred to as DR4 (direct-repeat with a 4 base pair spacer). *Abcb1* has three DR4 (AG(G/T)TCA) motifs approximately 8 kb upstream of its transcription start site [31]. In transgenic rodents expressing the human PXR, induction by specific PXR activators resulted in elevated *Abcb1* mRNA and transport activity [32,33]. This could suggest activation of PXR might be an approach to increase ABCB1-mediated BBB function.

The nuclear receptor constitutive androstane receptor (CAR) is activated by phenobarbital (PB) through a novel kinase cascade, but can also be activated by ligands such as the mouse specific CAR ligand: 1,4-bis[2-(3,5-dichloropyridyloxy)]benzene (TCPOBOP) or the human CAR ligand: 6-(4-chlorophenyl)imidazo[2,1-*b*][1,3]thiazole-5-carbaldehyde *O*-(3,4-dichlorobenzyl)oxine (CITCO). CAR activation, by either of the aforementioned mechanisms increases *Abcb1* and *Abcg2* mRNA and transport activity in rat and mouse brain capillaries in vitro and ex vivo [34]. AhR also functions in rat BBB and is capable of increasing ABCB1 and ABCG2 expression [35]. Induction of AhR using 2,3,7,8-tetrachlorodibenzo-*p*-dioxin (TCDD), an organic pollutant, increased expression and transport activity of ABCB1 and ABCG2 in the brain capillary membrane and was subsequently found to reduce brain accumulation of verapamil, an ABCB1 substrate [35]. 

The estrogen receptor has been implicated in regulating *Abcg2* transcription, specifically estrogen receptor β. Rat brain capillaries exposed to the estrogen receptor ligand, 17β-estradiol showed decreased ABCG2 levels and transport activity in an ex vivo rodent [36]. These results highlight the role of metabolites and xenobiotics in regulating drug-efflux transporters. A caveat is that most of these studies were conducted in rodent models and these pathways might not be conserved in humans. Incidentally, upregulation of ABCB1 and ABCG2 levels and function have been observed by some therapeutic agents (Table 1). For example, taxol has been shown to upregulate ABCB1 by PXR [37]. It remains to be determined how many of these agents (Table 1), use nuclear and/or hormone receptors, to upregulate ABCB1 and ABCG2.

Regulation of ABC transporters at the BBB by developmental pathways also occurs. For example, Wnt signaling appears important as ABCB1 has 7 consensus TCF/LEF binding motifs (CCTTTGA/TA/T) within its promoter. Although speculative, there is concordance between activation of the Wnt pathway and the BBB expression of ABCB1, which suggests that ABCB1 developmentally increased in the BBB to protect nascent CNS development. The Wnt pathway also modulates ABCB1 in other non-BBB ECs [17]. In intestinal EC lines, TCF4, a transcription factor of Wnt signaling, binds the human *ABCB1* gene promoter and activates transcription [59]. In the BBB, ABC transporter regulation by the Wnt pathway is conserved in brain ECs from both rats and humans (hCMEC/D3). Blocking the Wnt pathway “destruction complex” (composed of GSK3, Axin, PP2A, and CK1α) using various GSK3 inhibitors, activated the Wnt pathway, in both model systems producing upregulation of *ABCB1* mRNA, protein, and a concomitant increase in transport activity [60]. Conversely, shutting off the canonical Wnt pathway with the Wnt inhibitor, Dkk-1 (Dickkopf-related protein 1) strongly reduced ABCB1 and ABCG2 expression. Subsequent studies, using chromatin immunoprecipitation (ChiP) demonstrated that β-catenin bound to the *ABCB1* promoter upon activation of canonical Wnt signaling by WntA, correlated with increased *ABCB1* mRNA in hCMEC/D3 cells [61]. Furthermore, a non-canonical Wnt signaling pathway mediated by the RhoA/RhoA kinase (RhoAK) might regulate ABC transporters at the BBB as constitutive activation of RhoAK influenced β-catenin mediated transcription of *ABCB1* [61]. Together these results suggest Wnt signaling modulates ABCB1 expression and function through both canonical and non-canonical pathways. Modulation of the Wnt pathway might be an approach to alter the function of the BBB (discussed in Section 2.2.4). 

Another pathway important for BBB maintenance (described above) is the SHH pathway. A direct role for SHH signaling regulating ABC transporters at the BBB has not been demonstrated. However, some model systems suggest SHH modulates ABC transporters. For instance, activation of the SHH pathway in squamous cell carcinoma, and esophageal and prostate carcinoma cell lines all increased the mRNA and activity of both ABCB1 and ABCG2, as measured by transport using their respective substrates: docetaxel and methotrexate [62]. Likewise, in diffuse large B-cell lymphoma, SHH modulated ABCG2 expression through its main transcriptional activator, GLI1 [63]. A GLI1 binding site is in the *ABCG2* promoter and GLI1 knockdown resulted in a reduction in ABCG2 amount [64]. More definitive evidence for SHH signaling modulation of drug efflux transporters at the BBB awaits further study. 

#### 1.3.2. Inflammatory Signaling Pathways

In addition to ligand–receptor signaling described above, stress and inflammatory signaling pathways have been reported to modulate the drug-efflux transporters at the BBB. Stress induces secretion of inflammatory cytokines that result in a response mediated by neuroprotective proteins such as nuclear factor erythroid 2-related factor 2 (Nrf2), and nuclear factor κ-light-chain-enhancer of activated B cells (NF-κB). However, the reported effects of stress and inflammatory cytokines on ABC transporters at the BBB are complicated, and depend on the model system and exposure time used. 

ECs release inflammatory cytokines during cell injury. To mimic an insult with cytokines, rat brain ECs treated with the pro-inflammatory cytokines, tumor necrosis factor α (TNF-α) and interleukin-1β (IL-1β) exhibited a rapid decline in BBB integrity. This decline in BBB integrity was reversible, in contrast to interleukin-6, appears to permanently damage BBB integrity [65]. Subsequent study in an immortalized rat brain capillary EC line, GPNT, treated with TNF-α from 2 to 96 h showed induction of *Abcb1* mRNA over time, but resulted in no induction of ABCB1 protein [66], however, ABCB1 function measured by vinblastine accumulation, gradually increased over the 96 h treatment interval. A caveat: cell lines might not recapitulate primary cells because in another study, using primary rat brain capillaries, showed that *Abcb1* mRNA rapidly decreased after TNF-α exposure without altering ABCB1 protein level. This effect was reversible as the activity remained suppressed for 3 h post-treatment, but then gradually recovered [67]. The restoration of the protein during the recovery phase was attributed to activation of the NF-κB via the TNF-R1/NOS/PKC (Nitric oxide signaling/Protein kinase C) pathway. 

#### 1.3.3. Gender Bias and Age

Depending upon which transporter is studied, sexual dimorphism in transporter expression exists. However, in many cases, the mechanisms are unknown. Nonetheless, understanding how gender affects expression and function of these transporters may have important pharmacological and toxicological implications. For example, if one gender expresses lower transporter levels, possibly making this gender more prone to drug-induced side effects and toxicity, dosing adjustments may be necessary. 

To our knowledge, gender variation in ABC transporters at the BBB has not been reported. However, in non-CNS tissues, gender variation is well known. For example, hepatic ABCB1 was found to be 2.4-fold lower in females compared to males [68]. Conversely, ABCB1 protein levels are higher in female rats compared to male [69]. In rat, kidney ABCB1 level is affected by the menstrual cycle where the highest ABCB1 protein level correlated with the maximum progesterone level. The changes in expression might be related to transcriptional activation or suppression by steroid hormone receptors. For example, testosterone administration reduced ABCB1 protein expression in the liver that resulted in a reduction in hepatic doxorubicin clearance [70]. For ABCG2, hepatic ABCG2 protein levels are higher in male mice compared to female [71]. Sex-dependent physiological states (e.g., pregnancy, lactation, or virginity mice) were not found to contribute to the sexual dimorphism in one study [71]. In rats, *Abcg2* mRNA was higher in female brain and in male kidney. 

Age also impacted BBB drug-efflux protein expression. Reduced expression and function of ABC transporters, at the BBB, has been a proposed factor in susceptibility to age-associated diseases, such as neurodegenerative diseases. Positron emission tomography (PET) was used to measure uptake of the ABCB1 substrate ^11^C verapamil in the brain of 35 healthy men and women. Verapamil accumulated to higher levels in the older male cohort (ages 55–70 years) compared to young (ages 20–30 years) and middle age (40–50 years) [72]. This suggested ABCB1 function at the BBB declined with age only in men. In contrast, there was no association between age and verapamil accumulation in women. 

No age-related study on the BBB function of human ABCG2 has been reported to date. Nonetheless, studies on other animal models and tissues may still provide valuable insights. Monitoring hepatic *Abcg2* mRNA level, in rats, from birth to Day 60, revealed *Abcg2* mRNA peaked at seven days postnatal, followed by gradual decrease to Day 60 [73]. On Days 180 and 540, *Abcg2* mRNA was further decreased by 4–5 fold relative to Day 60. Overall, females tended to express higher *Abcg2* mRNA compared to males. This trend was not apparent at the protein level as males showed higher ABCG2 expression overall. 

#### 1.3.4. ABC Transporters and the Blood Brain Barrier in Cancer

The blood–brain barrier is altered in pathologies of the CNS including neurodegenerative diseases, epilepsy, brain cancers and metastatic brain tumors. Growing evidence indicates transporters at the BBB limit drug delivery to the brain, yet among treatments that target ABC transporters at the BBB, many of them have not been as successful as expected [74,75,76]. A possible explanation for this limited success is that drug resistant CNS tumors, expressing upregulated ABC transporters play a significant role in the diminished therapeutic response. 

Another important factor to consider is that the function of the BBB might be modified by tumor cells in the micro-environment. The TJ proteins occludin-1 and 3 are downregulated at the BBB in metastatic colon cancer and glioblastoma cells, resulting in a leaky BBB [77]. One potential mechanism to account for leaky BBB: tumors cells modify the vasculature by secreting angiogenic factors, such as VEGF (vascular endothelial growth factor) to induce formation of new blood vessels, which may not share the non-leaky properties of BBB. Another impediment to therapy is that tumor cells can, by expressing export transporters, form a barrier, the blood–tumor barrier (BTB) [78,79,80]. Consistent with this, human brain tumors of various grades and metastatic brain tumors express ABCB1 to create a BTB [81,82]. 

Tumors of embryonal origin, which includes (non)-small cell lung cancer (NSCLC/SCLC), melanoma, lung, and breast cancers, exhibit a high frequency of brain metastases [83]. Some metastatic brain tumors arising from primary NSCLC/SCLC, breast, and melanoma cells exhibit low ABCB1 expression, which correlates with increased permeability and drug uptake. For instance, paclitaxel accumulated to a greater extent in the metastatic gliomas, areas with low ABCB1 levels, compared to primary glioma as measured by MRI (magnetic resonance imaging) [83]. In brain tumor arising from metastatic breast cancer models, uptake of ABCB1 substrates paclitaxel and doxorubicin was higher than normal brain. Unexpectedly, the cytotoxic effects of these drugs remained relatively low [84]. Studies in metastatic tumors suggest that ABC transporters presence at the BBB and/or BTB of the primary tumors remain a major contributor of drug resistance and that residual ABC transporters function at the BTB is sufficient to limit drugs’ cytotoxic effects.

In the following sections, we review the potential contributions of ABCB1 and ABCG2 in gliomas and medulloblastoma. We will attempt to highlight the disease etiology and progression with respect to ABC transporters levels and functions and whether they affect treatment outcomes. 

## 2. ABC Transporters in Central Nervous System (CNS) Tumors

### 2.1. Gliomas

#### 2.1.1. Prognosis, Treatment, Cells of Origin

The progenitor cells of glial tumors can be from either the brain or the spinal cord. These include astrocytes that support neuron function, oligodendrocytes that myelinate neurons, microglia that remove dead neurons and pathogens, and ependymal cells that secrete CSF. Astrocytes and microglia contribute to the maintenance and function of the BBB as described earlier. The BBB function is compromised in certain gliomas. Gliomas are histologically classified into the following subtypes: astrocytoma, oligodendrocytoma, oligodendroglioma, and glioblastoma [85]. Gliomas are also divided into four grades based on their cell of origin, each with a different prognosis. Grade I glioma is called pilocytic astrocytoma, typically seen in pediatric population and is relatively benign. Grade II glioma, also called low-grade glioma, includes astrocytoma, oligodendroglioma, and mixed oligoastrocytoma, typically occurring in young adults. Grade II glioma can evolve into a more aggressive tumor. Grade III glioma, called malignant glioma, includes anaplasticastrocytoma, anaplastic with recurrent properties and oligodendroglioma, and anaplastic mixed oligoastrocytoma. These tumors have invasive properties and are more aggressive than grade II tumors. Grade IV glioma, referred to as glioblastoma multiforme (GBM) is the most aggressive and common primary brain tumor in adults. Treatment regimens for gliomas start by the surgical resection of as much tumor possible, followed by radiation therapy to eradicate remaining tumor cells. In grade III glioma, chemotherapy may be given after radiation therapy, whereas in GBM, due to its aggressive nature, chemotherapy is given in combination with radiation therapy. Recently, the 2016 World Health Organization Classification of tumors of CNS further categorized gliomas into subgroups based on distinct molecular signatures (e.g., IDH (isocitrate dehydrogenase)) mutation status that underlie tumorigenesis [85]. 

#### 2.1.2. Glioblastoma

Glioblastoma is the most lethal form of brain tumor in adults and about 50% of patients treated with the alkylating agent temozolomide (TMZ) are refractory to this treatment. In one of the largest phase II trials enrolling 162 anaplastic astrocytoma and glioblastoma patients, TMZ treatment resulted in a six-month overall survival rate of 75% with a median overall survival of 13.6 months, a marked improvement over the expected 6–9 months median survival. The overall response rate for this trial was 62% [86]. Furthermore, glioblastoma patients TMZ proved to be better than procarbazine monotherapy at first relapse [87]. In 2005, after a number of clinical trials found TMZ significantly increased survival, it was combined with radiotherapy to become the standard of care for glioblastoma treatment. Comparative analysis of large datasets, available from the Surveillance, Epidemiology, and End Results (SEER) Program, showed that TMZ addition to the radiation therapy regimen prolonged median survival times from 12.0 months to 14.2 months [88]. Prior to this modification, glioblastoma survival had not improved between 1980s and 2001. While TMZ has had a major impact on glioblastoma survival, improving elderly patient survival still remains a challenge. Their median survival is less than 15 months and many patients experience recurring tumors. In addition to *O*^6−^methylguanine methyltransferase (MGMT) status, treatment-refractory glioma stem cells (GSCs) are thought to be responsible for tumor recurrence. Compared to non-stem glioma cells, GSCs are more resistant to both chemotherapy and radiation. The resistance mechanisms include reduced cellular accumulation of chemotherapeutics, due to an increase in ABC efflux transporter expression, lack of or aberrant DNA repair mechanisms such as an increase in MGMT, which repairs TMZ-induced DNA damage, and upregulation of anti-apoptotic signals [89].

#### 2.1.3. Glioblastoma and ABCB1, ABCG2

In certain glioblastomas, high expression of *ABCB1* and *ABCG2* has been reported to associate with poor prognosis. A likely explanation is that transporters that limit drug accumulation are upregulated in the tumor cells thereby limiting drug uptake. Conceivably, ABCG2 might also have a biological role in tumor progression by affecting tumor stem cells. It is worth noting that ABCG2, while mainly discussed here for its function at the BBB as a drug transporter, is highly expressed in many stem cell compartments including GSCs. GSCs are considered to be more resistant to TMZ treatment; therefore, GSCs that survived the treatment are likely the cause of the recurrence often seen in patients.

**Figure 2:**
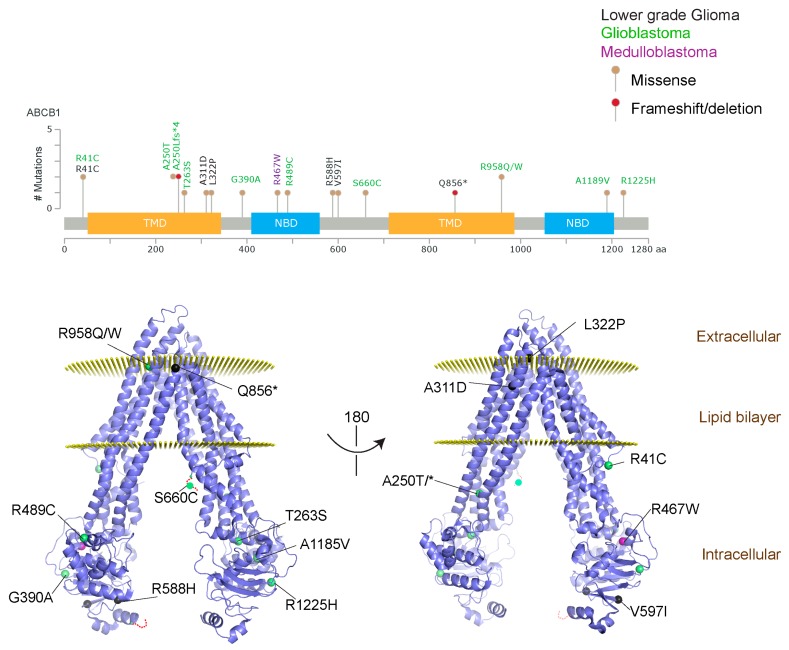
*ABCB1* mutations found in gliomas and medulloblastoma patients. (**Top**) Patient mutations in various regions of ABCB1 protein. Mutations are color-coded based on cancer and mutation type. Black (Lower-grade glioma), green (glioblastoma), purple (medulloblastoma). Brown (missense mutation), red (frameshift/deletion). (**Bottom**) ABCB1 patient mutations mapped on mouse ABCB1 (PDB (Protein Data Bank): 49qh). Spheres highlight the α-carbon of the corresponding amino acid. Disordered regions containing mutation sites that were not visible in the structure are represented as dashed lines in red. TMD, transmembrane domain; NBD, nucleotide-binding domain.

**Figure 3:**
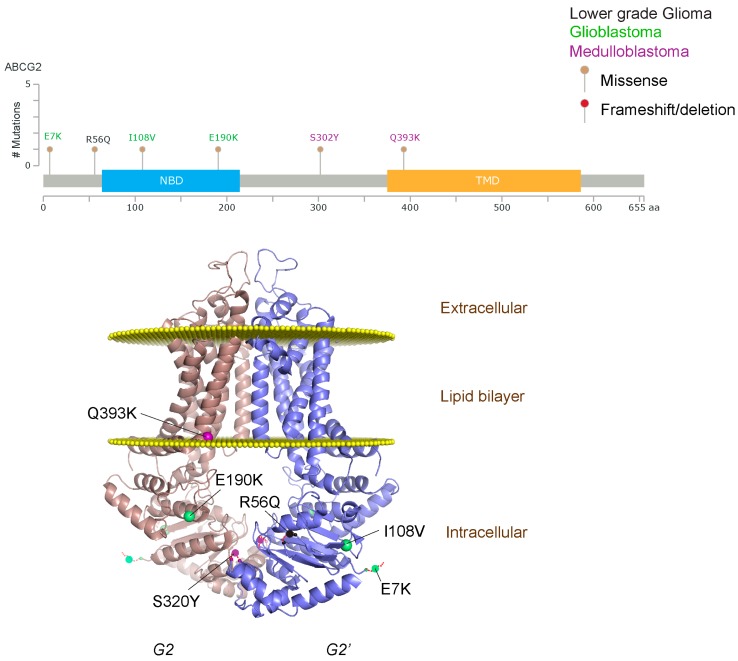
*ABCG2* mutations found in gliomas and medulloblastoma patients. (**Top**) Patient mutations in various regions of ABCG2 protein. Mutations are color-coded based on cancer and mutation type. Black (Lower-grade glioma), green (glioblastoma), purple (medulloblastoma). Brown (missense mutation), red (frameshift/deletion). (**Bottom**) *ABCG2* patient mutations mapped on human ABCG2 (PDB (protein data bank): 5nj3). Spheres highlight the α-carbon of the corresponding amino acid. Disordered regions containing mutation sites that were not visible in the structure are represented as dashed lines in red. G2 and G2’ refer to individual monomer polypeptides of dimeric ABCG2.

**Figure 4:**
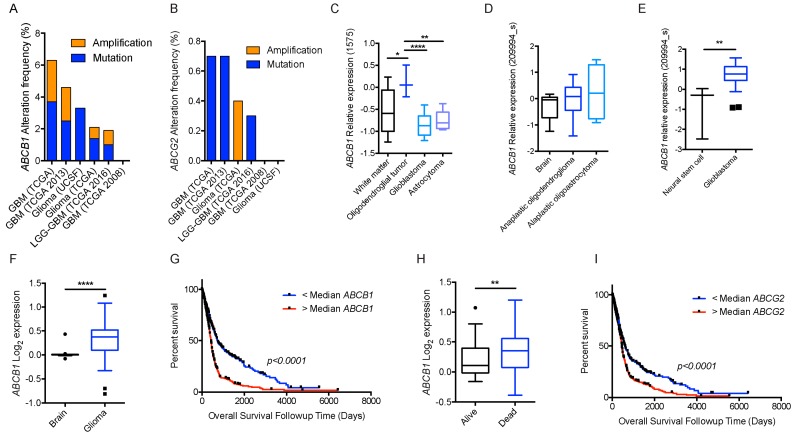
BBB ABC transporters and gliomas. (**A**) Frequency of *ABCB1*; and (**B**) frequency of *ABCG2* mutations and amplifications depicted glioma patient cohort data deposited in cBioportal. *ABCB1* RNA expression data curated from the Oncomine database in normal tissue as described and different subtypes of gliomas plotted on Tukey plots from: (**C**) Shai [90]; (**D**) French [91]; and (**E**) Lee studies [92]. (**F**) DNA copy number levels of *ABCB1* in brain and glioma samples in TCGA study. (**G**) Overall survival of glioma patients with *ABCB1* DNA copy number higher (red) or lower (blue) than median is shown. (**H**) DNA copy number levels from Astrocytoma in Kotliarov study (Oncomine) for patients who were alive or dead at three-year follow up time point. (**I**) Overall survival of glioma patients with *ABCG2* DNA copy number higher (red) or lower (blue) than median. *p* values were examined by 2-Way ANOVA for (**C**) and by two-tailed student *t*-test for (**D**–**F**,**H**), *, *p* < 0.05; **, *p* < 0.01; ****, *p* < 0.0001. Tukey plot whiskers (fences) extend to the most extreme data point that is no more than 1.5 times the interquartile range. The individually plotted values are outliers and the boxed limits cover the lower and upper quartiles with a line representing the median.

We took advantage of the publicly available databases to evaluate whether *ABCB1* and *ABCG2* genes were altered, by mutation or amplification, in CNS tumors, glioblastoma and medulloblastoma (the most common pediatric CNS tumor- will be described in details later). Interrogating the cBioportal (http://www.cbioportal.org/, accessed on 23 July 2017) and PeCan data portals (https://pecan.stjude.org/home, accessed on 23 July 2017), 10 mutations in *ABCB1* and three in *ABCG2* genes in were identified in glioblastoma alone (Figure 2 and Figure 3). Although the functional impact of these mutations has not been formally tested, it is worth noting that one mutation in *ABCB1* produces a frameshift and an early stop codon, suggesting that this particular allele is an inactivating mutation. Interestingly, most of the mutations are clustered in the nucleotide-binding domains (NBDs) of both ABCG2 and ABCB1 (Figure 2 and Figure 3). ABC transporters use the binding and hydrolysis of ATP molecules at NBDs as the energy to induce both the conformational changes that facilitate substrate translocation and that re-set the transporters back to their original conformation. Mutations in the NBD can de-stabilize ABC transporters. Further studies are needed to determine the impact of these mutations on expression and transport function of these proteins. cBioportal also compiled data on the genomic amplification status of these genes. For *ABCB1*, as much as ~4% of the glioblastoma patients carried mutations and another 2.5% of the patients carried amplification of the gene (Figure 4A, TCGA). TCGA is the largest dataset available (500+ samples) so this might account for its power to discover genomic alterations compared to other studies. Unlike *ABCB1*, alterations in the *ABCG2* gene rarely appear, with less than 1% of patients carrying either mutations or amplification. 

Neither ABCB1 nor ABCG2 expression was detected in the brain parenchyma under normal conditions, specifically astrocytes and glial cells, which are cells of origin for some gliomas. Interestingly, *ABCB1* mRNA levels were higher in some gliomas than the control brain (Oncomine.org, accessed on 23 July 2017; Figure 4C–E) [90,91,92]. This increase in mRNA corresponded with a higher DNA copy number (TCGA dataset, Figure 4F). Of note, higher than median *ABCB1* DNA copy number was also associated with significantly shorter survival in this dataset (Figure 4G). In another dataset from astrocytoma (the Kotliarov study [93]) patients who died by three-year follow-up had a significantly higher DNA copy number (Figure 4H). Although there was no difference in *ABCG2* copy number in normal brain and glioma samples in the TCGA study, higher than median *ABCG2* copy number was still significantly associated with poor overall survival (Figure 4I). It is important to note that mRNA levels of these genes were not significantly associated with overall survival status.

#### 2.1.4. ABCB1 and ABCG2: Impact upon Temozolomide (TMZ) Therapy

The alkylating agent TMZ is the drug of choice in treating glioblastoma and the contribution of ABCB1 and ABCG2 to its efficacy has been widely studied. One study found increased TMZ accumulation in the tumors of mouse GBM model when the dual ABCB1/ABCG2 inhibitor, elacridar was used or in mice that were deficient for both genes, suggesting that TMZ is extruded from the brain by these transporters [52]. They also found that ABCG2 was likely to provide additional protection due to its high expression in tumor capillaries (blood–tumor barrier). 

ABCG2 protein has been detected in both mouse models of glioblastoma and human glioblastoma samples by IHC (Immunohistochemistry) [52]. However, the intensity of immune-staining was heterogeneous, which might reflect the GSCs, noted for their high expression of ABCG2. Importantly, GSCs reportedly are refractory to TMZ treatment compared to non-GSC glioblastoma cells. Another study evaluated ABCG2 status in 33 human primary glial tumors from patients with refractory epilepsy [94]. Very robust ABCG2 expression was detected in anaplastic astrocytoma and glioblastoma patient samples by Western blot and modest level in grade II astrocytoma. Unexpectedly, IHC revealed only ABCG2 staining in the tumors microvessels, but not in the tumor cells. The authors proposed that the apparent increase in tumor ABCG2 expression was actually due to increased expression in the tumor vasculature, not the tumor. However, these studies overlooked the possibility that a rare GSC expressing ABCG2 can affect treatment outcome. In glioblastoma cell lines, TMZ treatment increases the side population (SP) [95], a phenotype characterized by high ABCG2 function and an enrichment of stem cells. Incidentally, TMZ has also been reported to increase ABCB1 expression and transcription via EGFR (Epidermal Growth Factor Receptor). These SP cells were more resistant to TMZ suggesting that ABCG2 might have a role in TMZ sensitivity. Expression of ABCG2 appears related to survival in glioblastoma patients. In a study of 50 human glioblastoma patients high ABCG2 levels were associated with much worse survival with an adjusted hazard ratio of 2.35 [96]. Furthermore, treating the patient-derived glioblastoma tumor spheres with an ABCG2 inhibitor reduced self-renewal of these cells suggesting that ABCG2 is important for maintaining their stemness [97]. Despite these findings, some studies showed that ABCG2 knockdown does not affect TMZ sensitivity in glioma cell lines. This discrepancy in the role of ABCG2 in affecting TMZ sensitivity might be due to inherent differences between in vivo and in vitro model systems. 

In contrast to ABCG2, ABCB1 protein amounts in 60 CNS tumor samples, including several gliomas, revealed no significant change in expression compared to the normal brain parenchyma. However, some TMZ-resistant glioblastoma cells exhibit increased levels of ABCB1 and ABCG2. In these cells, the SHH pathway was upregulated as a result of PTCH1 repression by the microRNA, miR-9. Expression of miR-9, in U87 and T98G glioblastoma cell lines, was associated with increased amounts of *ABCB1* and *ABCG2* mRNA and protein [98]. This microRNA might mediate *ABCB1* and *ABCG2* upregulation. Strikingly, a link between ABCB1 and glioblastoma treatment efficacy came from an analysis of *ABCB1* SNPs in TMZ-treated glioblastoma patients. In this cohort of 112 patients, three SNPs *C*1236*T*, *G*2677*T*, and *C*3435*T* were assessed for an association with the two-year overall survival [40]. Multivariate analysis revealed that the *C*1236 genotype was a predictive marker for survival. Patients with the 1236*C/C* genotype displayed a better two-year survival (37%), than patients with *T/C* and *T/T*, which had only 8% and 10% chance of survival, respectively (*p* = 0.02). The *C*1236*T* is reported to be an activating SNP, where ABCB1 transport function was increased for several chemotherapeutics. 

#### 2.1.5. Improving Glioblastoma Therapy by Altering Blood–Tumor Barrier (BTB) and BBB/ABC Transporter Function

As outlined above (and listed in Table 1), growing evidence highlights how glioblastoma treatment outcomes, in patients and model systems, are impacted by ABC transporters at the BBB. As such, inhibition of these transporters is an attractive strategy to improve therapeutic efficacy. Given the prominence and substrate overlap between the two major BBB ABC transporters discussed here, dual inhibition of ABCB1 and ABCG2 might be necessary to fully block drug exit from the CNS. For example, the ABCB1 specific inhibitor, zosuquidar (LY335979) enhanced sunitinib brain concentration in mice, but not to the same degree as the dual ABCB1/ABCG2 inhibitor, elacridar [57]. Elacridar was also effective in increasing brain exposure of dasatinib, a tyrosine kinase inhibitor, currently in clinical trials in glioblastoma cell lines and preclinical models [55]. In lower-grade glioma preclinical model, elacridar enhanced the brain penetration and efficacy of TMZ and PARP inhibitor ABT-888 (Abbott, now AbbVie) [52]. 

In addition to utilizing dual ABCB1 and ABCG2 inhibitors, an assessment of how the BBB impacts a drug’s efficacy can guide the development of better therapeutics to improve clinical outcome. For example, Salphati and coworkers found that the PI3K (Phosphoinositide 3-kinase) inhibitor, GDC-0941, was effective in reducing the tumor volume in the U87 orthotopic glioblastoma mouse model, but not in the GS2 model [99]. The primary difference is that the former lacked an intact BBB, whereas in the latter the BBB was intact; and an important consideration was that GDC-0941 was a substrate of both ABCB1 and ABCG2 [99,100]. By improving the physicochemical properties of GDC-0941, such that it bypassed ABCB1 and ABCG2 efflux, GNE-317 (an improved version of GDC-0941) effectively decreased tumor growth in all glioblastoma models tested, regardless of the BBB integrity and function [99]. Most recently, the authors have demonstrated the effectiveness of GNE-317 for the treatment of brain metastasis arising from melanoma tumors [101]. Furthermore, tumors with an increased BTB permeability also responded well to these drugs, even if they were ABCB1 and ABCG2 substrates. Importantly, metastatic brain tumors with an intact BTB only responded to GNE-317 as it evades the BTB efflux transporters [101]. In cases where drug modification or ABC transporter inhibition is not feasible, alternative approaches might be used: either a drug delivery system such as nanoparticles or CNS-invading pathogens [102].

In total, these studies demonstrate that both BBB integrity and its resident transporters are the key obstacles that must be overcome to achieve efficacious treatment. Targeting the BBB integrity and improving the properties of inhibitors may aid in BBB penetration and improve clinical outcomes, not only for glioblastoma, but also metastatic brain tumors.

### 2.2. Medulloblastoma

#### 2.2.1. Medulloblastoma Overview

Medulloblastoma (MB) is the most frequent malignant pediatric brain cancer, accounting for about 20% of all childhood brain tumors [103]. Historically, it was stratified based on histological features such as: desmoplastic/nodular, large-cell and anaplastic [104,105,106]. The advent of whole genome sequencing methods permitted the development of a molecular MB classification scheme, compromised of four distinct subgroups: Wingless (Wnt), sonic hedgehog (SHH), Group 3, and Group 4 [107,104]. Each subgroup exhibits signature molecular and transcriptomic features suggesting a distinct cell of origin for each subgroup [108]. These subgroups also have distinct histology, demographics, and clinical outcomes, the later due, in part, to different therapy. 

MB therapy typically involves surgical resection, followed by cranio-spinal radiation and chemotherapy. MB can metastasize to the pial surface of the spinal cord and brain. MB subgroup does not typically change at recurrence. In two independent and distinct cohorts, the molecular signature, therefore, subgroup affiliations, of recurrent and metastatic MB tumors remained the same [109]. In contrast, recurring and metastatic gliboblastoma tumors frequently shifts to heterogeneous subclass of tumors [110,111]. MB patients with metastatic tumors are deemed high-risk and receive cranio-spinal radiation of the entire nervous system instead of just the primary tumor site. While frontline treatments of MB have largely been successful, with about 70% survival rate amongst average-risk patients, the long-term side effects include hearing loss, a result of both radiation treatment and platinum chemotherapy, and most severely, neurocognitive impairments. A study of 380, adult survivors of childhood MB and PNET (primitive neuroectodermal tumor) showed 37.4% incidence of hearing loss, and 72.3% of survivors experiencing problems of balance and coordination as well as tremors [112]. Lower educational attainment and social independence were also reported to be pronounced risks associated with current MB treatments [112]. Additional markers might also guide more effective treatment. For instance, β-catenin status is a marker for favorable of outcome in pediatric medulloblastoma [113,114]. For instance, pediatric cases with positive nuclear β-catenin staining exhibited significantly better survival than those with negative nuclear staining and positive cytoplasmic and membrane β-catenin staining. Such predictive markers could also guide treatment choice to reduce the adverse effects of conventional therapies [113]. While lowering the dosage can reduce the side effects of cranio-spinal radiation and chemotherapies on neurocognitive functions [115], the risk in a less intense therapy is a greater likelihood of an unfavorable outcome. Therefore, an improved understanding of the disease at the molecular level will help contribute to the refinement of the current therapies. 

#### 2.2.2. Etiology of Medulloblastoma

As embryonal tumors with primitive characteristics, MB have the ability to differentiate into multiple lineages [85]. About 30% of medulloblastoma tumors belong to the sonic hedgehog (SHH) subgroup. They arise as a result of over-proliferation of granule neuron progenitors (GNPs) [116,117]. The GNPs arise from a dorsal hindbrain structure called the rhombic lip [118]. They expand postnatally, receiving proliferative cues from the sonic hedgehog mitogen secreted by purkinje cells [119]. Expansion of GNPs is followed by their outward migration to the external germinal/granule layer (EGL) then inwardly along the radial fibers of Bergmann glial cells to form now differentiated neurons in the inner granule layer (IGL). The precise timing of GNPs expansion and migration ensure proper development of the cerebellum [120]. 

Aberrant SHH signaling was first linked to medulloblastoma as Gorlin’s syndrome patients, harboring a mutated *PTCH1* gene, exhibited an increased incidence of medulloblastoma [121]. Heterozygous *Ptch1* mice developed tumors on the surface of the cerebellum, which closely resemble human medulloblastoma [122]. GNP expansion occurs with precise spatiotemporal regulation. This cascade can be disrupted by *PTCH1* inactivation by mutation or loss, SMO mutation, and *GLI2* amplification, some of these are known drivers of SHH-MB [123]. Commitment to the GNP lineage is a crucial determinant of SHH-MB tumorigenesis as smoothened activation in astrocyte (*Gfap^+^*) or oligodendrocyte (*Olig^+^*) progenitors gave rise to MB with 100% penetrance and with similar histology and expression signatures as one derived from GNPs [124]. SHH-MB can also arise from GNPs derived from cochlear nuclei of the brain stem [125]. Complete loss of PTCH1 function is not immediately tumor-forming suggesting, temporal acquisition of genetic lesions contributes to the development of SHH-MB. Enforced expression of *MYCN* proto-oncogene (a known GLI1 target gene) in GNPs isolated from *Trp53*-null mice results in tumors that closely resemble *Ptch1* sonic hedgehog tumors with increased penetrance [126,127]. 

In contrast, overexpression of *c-MYC* in GNPs (isolated from *Trp53*-null mice) enabled the development of the first engineered murine model that reproduced the human Group 3 medulloblastoma [127]. Enforced expression of a partially stabilized T58A MYC mutant with a dominant negative *Trp53* in neural stem cells also faithfully recapitulated Group 3 medulloblastoma [128]. Interestingly, activation of GABAergic and photoreceptor pathways is a signature of Group 3 tumors [129,123]. *MYC* amplification occurs in about 10–20% of Group 3, the highest among the subgroups [104,105]. Interaction of MYC with a binding partner, MIZ1 (MYC-interacting zinc finger protein 1) is required for development of Group 3 tumors [130]. Expression of MYC V394D mutant, with attenuated binding to MIZ1, delayed disease onset and produced tumors with transcriptional profiles that are distinct from both Group 3 and SHH-MB. 

The cells of origin for Group 4 are not known to date. Targeted expression of partially stabilized version of *MYCN* (T28A) in neural stem cells isolated from P0 cerebella resulted in tumors with a Group 4 signature [131,132]. At the transcriptional level, Group 4 tumors, are characterized by activation of neuronal and glutaminergic pathways [123,105]. Group 4 tumors show in 80% of the cases of aberration at i17q (isochromosome 17q). Mutations in chromatin modifiers are common among Group 3 and Group 4, suggesting a shared mechanism for transformation [129]. Efforts to better delineate Group 3 and Group 4 signatures include integration of genomic, transcriptomic and epigenomic analyses [133,134]. 

In contrast to Group 3 and 4 tumors, which arise from GNPs or neural stem cell populations, Wnt tumors arise from progenitors in the lower rhombic lip of the dorsal brainstem [108]. Similar to SHH tumors, Wnt MB result from constitutively active Wnt signaling. Activating mutations in the *CTNNB1* gene have been mapped as a strong driver of Wnt-MB with a mutation rate of 85–95% [135]. Wnt tumors are very responsive therapeutically (see below). 

#### 2.2.3. Demographics, Treatments and Clinical Outcome

Medulloblastoma in general are more commonly found in males than females with an overall ratio of 1.5:1 [136,137]. Wnt and SHH tumors have balanced sex ratios whereas there Group 3 and 4 tumors has 2:1 male:female ratios [136,137]. The mechanisms underlying the slight gender bias and whether it contributes to clinical outcome remain unknown. Wnt tumors, which are rarely metastatic, account for about 10% of all MB tumors. Medulloblastoma affects infants, children, and adults. The overall 5 years survival rate for Wnt MB is 95% [113,117]. Although Wnt-MB have mutant *TP53*, it does not appear tocontribute to clinical outcome (overall 5 year survival rates of 90% and 97% for WT and mutated *TP53* respectively) [138]. 

SHH tumors are more prevalent in infants and adults compared to children. The risk stratification of SHH tumors is influenced by their *TP53* status with higher risk associated with p53 germline or somatic mutations. Multivariate analysis deemed *TP53* mutation status as the most important risk factor in SHH-MB. Patients without *TP53* mutations have 5-year overall survival rate of 81% compared to 41% for mutated *TP53* high-risk patients [138]. *PTCH1* and *SUFU* germline mutations are frequently found in infants. Surprisingly infants have the best prognosis compared to children and adults. However, *TP53* mutation in children is frequently accompanied with *GLI2* and *MYCN* amplification, conferring a poor clinical outcome. Adult SHH tumors carry the most mutational burden which includes *PTCH*, *SMO*, and *TERT* [139,133]. SHH tumors can metastasize, but recurrence is usually local [109]. FDA (Food and Drug Administration)-approved SMO inhibitors have been included in clinical trials for SHH-driven tumors and they show moderate efficacy, but often transient due to the rise of mutants resistant to SMO inhibition [140,141,142]. 

Group 3 tumors, which account for 25% of all MB tumors, are rarely found in adults. The overall 5 years survival rate of Group 3 patients is 50% [135]. The outcome depends on *MYC* status and whether metastases are present at the time of diagnosis with about 50% of cases at the time of diagnosis harboring metastases. Non-metastatic and absence of *MYC* amplification patients are considered standard risk with 75–90% survival while metastatic tumors, especially with *MYC* amplification considered very high risk (<50% survival) [143]. With faithful model systems available, promising therapeutics targets have been identified for this MB subgroup and are actively being pursued which includes bromodomain inhibitors, PI3K and histone deactylase (HDAC) inhibitors, pemetrexed, gemcitabine, and CDK (cyclin-dependent kinase) 4/6 inhibitors [144,145].

The remaining 35% of MB tumors belongs to Group 4, which affects all ages, but predominates in children age 3–16 with a slight gender bias towards males. About 35–40% of Group 4 metastatic cases are presented at diagnosis. Gain of chr17 or loss or chr11 is associated with a favorable outcome [117]. Non-metastatic Group 4 patients have a survival rate of 90% [146]. The lack of preclinical model for Group 4 tumors hinders the development of a targeted therapy. 

Overall, the subclassification of medulloblastoma, using both clinical and molecular signatures, has proven beneficial in guiding therapy leading to improved prognosis. Nonetheless, a challenge remains for MB patients who do not respond to current therapies. This year, a number of groups proposed further refinements in the classification of medulloblastoma subtypes [134,147]. These in-depth stratifications can solve the heterogeneity within subgroups and is crucial in determining when de-escalation therapy might be beneficial; for example, in subtypes with an excellent prognosis such as Wnt and a subset of the SHH medulloblastoma. For MB patients, that are refractory to current therapies, an improved stratification might guide in the development and selection of therapies that target implicated disease driver pathways, an approach that should improve clinical outcomes.

#### 2.2.4. ABC Transporters and Medulloblastoma

A major challenge to efficacious medulloblastoma therapy is identifying drugs that are not just effectious in cell line models, but also must also be capable of penetrating the blood–brain barrier (BBB). Furthermore, if a drug is able to cross the BBB, the next hurdle is to effectively penetrate the tumor (the blood–tumor barrier, BTB). Drug-efflux transporters, inherently expressed in tumors may restrict drug penetration or tumors acquire increased expression through therapy. Standard chemotherapeutics used in MB treatment include combinations of vincristine, carmustine, procarbazine, prednisone, cyclophosphamide, CCNU (chloroethyl-cyclohexyl-nitrosourea), cisplatin, and carboplatin, many of which are reported to be substrates of ABCB1 and/or ABCG2 in various cell lines and in vivo murine models [148,149] (Table 1). For example, Saridegib (IPI-926), an FDA-approved SMO inhibitor, showed survival advantage for an extremely aggressive SHH-MB mouse model (*Ptc1-null* GNPs) [150]. Nonetheless, a fraction of these tumors survived despite the initial response to saridegib. This impaired response was attributed to ABCB1, whose expression unexpectedly increased during the 6 weeks of saridegib treatment. Treatment of tumors with an ABCB1 inhibitor, verapamil, enhanced saridegib’s ability to reduce GLI1 level even after extended treatment periods, highlighting the role of ABCB1 in mediating tumor growth in this SHH-MB model. ABCB1 expression is also associated with high-risk MB. Cells lines established from primary and recurrent tumors display high ABCB1 expression and activity [38]. Inhibition of ABCB1 by verapamil and vardenafil sensitize to etoposide, implicating the role of ABCB1 in etoposide export in these medulloblastoma model systems [38].

**Figure 5:**
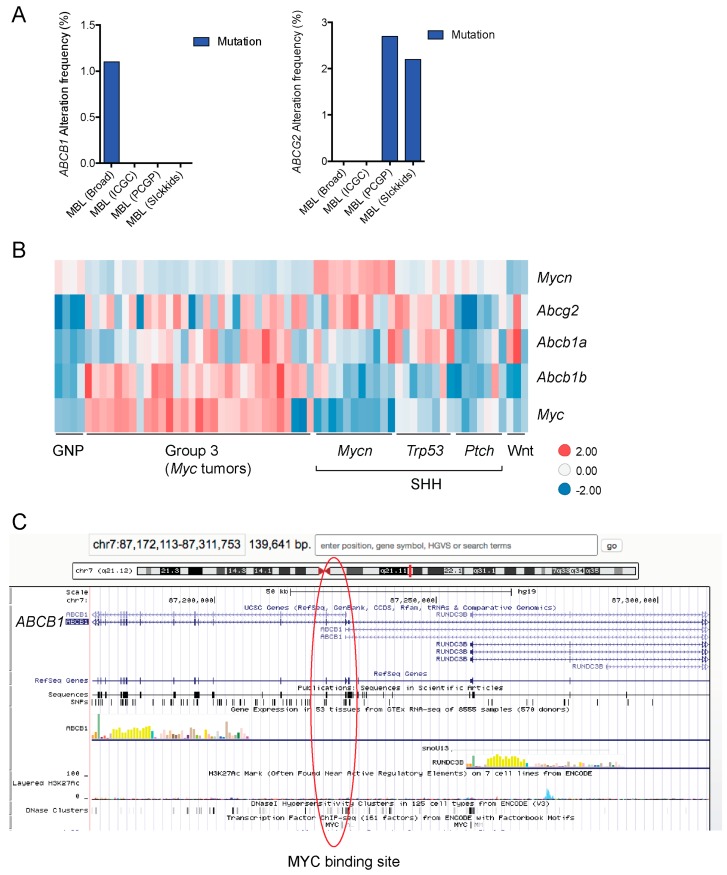
BBB ABC transporters in medulloblastoma. (**A**) Frequency of *ABCB1* and *ABCG2* mutations in medulloblastoma patient cohorts data deposited in cBioportal (*n* = 37 for PCGP (pediatric cancer genome project, *n* = 46 for Sickkids, *n* = 92 for broad, *n* = 125 for ICGC (International Cancer Genome Consortium); Gene expression data from these cohorts were not available for analysis. (**B**) Expression data for mouse granule neuron progenitor and medulloblastoma tumors from publicly available dataset, GSE33199 [127] shown in a heatmap. (**C**) *ABCB1* MYC binding site identified by ENCODE ChIP assays is highlighted in red circle (UCSC genome browser). MBL, medulloblastoma.

In addition to high ABC transporter expression in tumors, gain of function mutations of these transporters might account for impaired responses to chemotherapy. To evaluate *ABCB1* and *ABCG*2 for mutations in patients, we used the cBioportal platform with cohorts from four studies at the Broad, ICGC (international cancer genome consortium) , PCGP(pediatric cancer genome project), and Sickkids. Although some overlap occurs among the cohorts, somatic mutations in *ABCG2* and *ABCB1* are rare and only observed at a frequency of 1–2% (Figure 5). The somatic mutations in MB patients were mapped and the α-carbon of the mutant residue is highlighted in purple in the x-ray crystal and cryo-EM (electron-microscopy) structure of mouse ABCB1 and human ABCG2 respectively (Figure 2 and Figure 3). Intriguingly, R467W mutation in ABCB1 lies within the nucleotide-binding domain (NBD), which might alter the nucleotide-dependent activity of the transporter (Figure 2). In contrast, no mutation residing within the NBD was discovered in ABCG2. However, the S302Y mutation is present at the intervening region connecting the NBD and transmembrane domain (TMD) (Figure 3), whereas Q393K lies within the lipid bilayer interphase (Figure 3). Studies to determine whether these mutations affect ABCB1 and ABCG2 expression and function will provide valuable insight into the possible role of these ABC transporters in either the pathogenesis of MB or response to therapy.

Transcriptional regulation of ABCB1 and ABCG2 is another avenue by which these transporters. Proto-oncogenes *MYC* and *MYCN*, both of which are commonly amplified in MB have displayed regulatory effects on *ABCB1* and *ABCG2* in various models. In CD34^+^ chronic myeloid leukemia (CML) hematopoietic progenitor cells, ABCG2 expression is positively regulated by MYC [151]. In neuroblastoma cohorts, ChIP analyses revealed that MYC and MYCN can bind at *ABCG2* promoter, demonstrating a correlation between level of these transcription factors and *ABCG2* mRNA [152]. In the context of medulloblastoma, work by Ingram and coworkers showed *MYC* and *ABCG2* are highly expressed together at the mRNA and protein levels in 10 Gy radiation-resistant MB cells (DAOY and UW228 parental line) [153]. Whether MYC is the one responsible for increasing ABCG2 expression was not explored in this study. Nonetheless, qRT-PCR analysis of tumors resected from pediatric MB patients showed *ABCG2* is commonly expressed in pediatric MB tumors and its expression is higher than normal cerebellum [153]. When we assessed publically available mouse medulloblastoma gene expression data [127], a similar trend revealed higher *ABCG2* expression in the majority of tumors compared to the cell of origin for MB, the GNPs (Figure 5B). Specifically, high *ABCG2* expression clustered with Group 3 tumors, which was engineered by enforced expression of *MYC*, likely hinting that *MYC* might be responsible for the high *ABCG2* expression as has been previously reported in CML and neuroblastoma. Interestingly, ABCB1 expression was also high in Group 3 tumors (Figure 5B). MYC might also transcriptionally activate *ABCB1* as a candidate MYC binding site is reported within the *ABCB1* gene (Figure 5C). Overall, given that both of ABCB1 and ABCG2 are upregulated by MYC, MYCN, and Wnt signaling, it is conceivable that they could play a role in not just MB progression, but response to certain drug therapies and by altering their expression affect these regulators, to improve chemotherapeutic response. 

#### 2.2.5. Improving Medulloblastoma Therapy by Concurrently Altering the BTB and BBB by Targeting ABC Transporter Function

The low survival rate of Group 3 MB patients indicates an unmet need for an effective therapeutic intervention. Our group first reported that ABC transporter present in the tumor contributes to resistance to chemotherapy in medulloblastoma patients. Specifically, *Abcg2* was found to be highly expressed in the murine model of Group 3, (developed enforced expression of MYC in GNPs isolated from *Trp53^−/−^*; *Cdkn2c^−^*^/*−*^ mice) which has gene expression signatures that resemble the human counterpart [46]. RNA expression of *ABCG2* is high in both human and murine Group 3 MB. These findings supported the idea that Group 3 MB has intrinsically high levels of ABCG2. In the Group 3 model, in vitro studies (with tumorspheres) suggested ABCG2 inhibition might improve the therapeutic response. ABCG2 functions at the plasma membrane where it exports topotecan, one of the drugs used for recurrent MB in Group 3 patients [154]. In vivo, we found that the combination of topotecan with the well-tolerated Ko143, a dual inhibitor of ABCB1 and ABCG2, reduced tumor growth by more than 240% compared to single agents treatment, translating to an increase in survival from 17 to 28 days [46]. 

A recent study showed altering BBB integrity could improve therapeutic endpoint [155]. Phoenix and coworkers observed significantly more frequent CNS hemorrhaging in Wnt subgroup tumors in both patients and a Wnt mouse model (*Blbp-Cre^+/−^; Ctnnb1^+/lox(ex3)^; Trp53^+/flx^* mouse model) compared to other subgroups. Both human and mouse Wnt tumors had 3–4 times marker stainings that are indicative of aberrant vasculature and compromised BBB. Transcriptomic analysis of EC from Wnt tumors revealed downregulation of typical BBB endothelial cell markers such as CLDN5 (Claudin5) and SLC2A1 (Solute carrier family 2 member 1), which was confirmed by an accumulation of Tetramethylrhodamine-dextran(TMR-dextran) in Wnt tumors compared to SHH tumors, signifying that Wnt tumors have a leaky BBB. This BBB leakiness, specific to Wnt MB tumors, is a result of high levels of soluble Wnt pathway inhibitors, Dickkopf 1 (Dkk1) and Wnt inhibitor factor 1 (Wif1), both of which were absent in SHH and Group 3 tumors. The authors reasoned that the high level of Wnt inhibitor is a result of constitutive paracrine Wnt signaling induced by β-catenin mutation, secreting Wnt inhibitors as a negative feedback loop. Indeed, nuclear LEF1 was specifically absent in the endothelial cells of Wnt tumors, indicating Wnt signaling was not active within the tumor, but active everywhere else. To test the role of Wnt signaling in regulating BBB phenotype in medullobastoma, the authors enforced expression of Wnt inhibitors, Wif1 and Dkk1 in mouse SHH-MB model and enforced activation of Wnt signaling in Wnt MB tumor by expressing Wnt7a agonist. Restoration of endothelial Wnt signaling in Wnt-MB tumor restored BBB integrity. Inhibition of Wnt signaling in SHH tumor significantly increased BBB permeability. These manipulations translated into changes in drug penetrance. Tumor concentrations of vincristine were higher in Wnt tumors with the leaky BBB compared to the BBB from tumors engineered to express a Wnt7a agonist. Together, this study highlights the BBB as the major barrier for drug penetration into the brain parenchyma in medulloblastoma.

**Figure 6:**
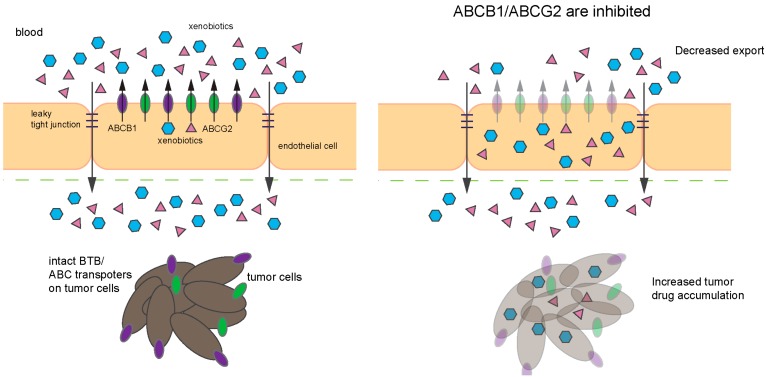
Schematic depicting the effect of ABCB1/ABCG2 inhibitors. (**Left**) Compromised BBB (tight junctions (TJ) impairment, etc.) in certain tumors, such as glioblastoma, increases drug penetration into brain parenchyma. However, ABC transporters (purple, ABCB1; and green, ABCG2) expressed in the tumor cells may extrude chemotherapeutic drugs, thus limiting the drugs effectiveness. (**Right**) Inhibition of ABC transporters with or without compromised BBB will not only increase drug accumulation in the brain parenchyma but also allow the drugs to accumulate in the tumor and tumor stem cells.

In the context of ABC transporters, endothelial cells isolated from Wnt-MB tumors exhibited significant reduction in *Abcb1* mRNA [155]. As previously described, interplay exists between BBB, ABCG2 and Wnt signaling where β-catenin activation upregulates ABCG2 level. Since ABCB1 and ABCG2 are the major drug-efflux transporters at the BBB, that are highly expressed in Group 3 MB and *MYCN*-amplified SHH-MB (Figure 4), it would be beneficial to determine if these transporters are expressed at the tumor ECs of the various MB subgroups. Finally, given the availability of SHH and Group 3 preclinical models, pre-treatment or combination of ABCB1 and ABCG2 inhibitors with frontline therapies is worth investigating as it could increase penetration and cytotoxic effects of drugs and improve clinical outcomes (Figure 6). 

## 3. Perspective

Therapeutic targeting of CNS cancers remains a challenge. Both the BBB and the BTB have impeded success; our understanding of the latter is nascent and up until recently BTB has not been a point of focus because the BBB seemed to be such a prominent obstacle. Currently, we have only a rudimentary understanding of the BTB and which transporters contribute to therapeutic failure. This is a future avenue of exploration. An ideal current scenario, to advance therapy, might be combination therapy, using an inhibitor that blocked or suppressed the function of both ABCG2 and ABCB1 (depicted in Figure 6). 

## Figures and Tables

**Table 1:** List of chemotherapeutic reagents currently being used for treatment of gliomas and medulloblastoma.

Drug	Type of Drug/Cancer	Transporter Implicated in Drug Resistance	Model System
Temozolomide (TMZ) 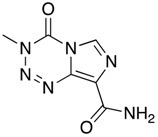	Alkylating agent GBM, MB [38]	ABCB1	*Mdr1a^−/−^* mice accumulated higher TMZ in the brain compared to *Mdr1a^+/+^* mice [39] (*Mdrla = Abcb1a*).
		ABCB1	GBM patients with C/C variant at amino acid 1236 of ABCB1 respond better to TMZ than patients with C/T and T/T variants, which results in higher ABCB1 expression [40].
		ABCG2	GBM cell lines (U251, A271), cell lines derived from primary tumors (MZ-327, MZ-18), and cell lines from recurrent grade IV tumors (MZ-256, MZ-304) were treated with TMZ. Cell survival was measured by MTT and trypan blue assays. Cells treated with reversan, which inhibits both ABCB1 and ABCG2 showed increased sensitivity to TMZ [41].
		ABCG2	GBM cells line (A172, U87, and U373) and neurospheres derived from primary GBM showed increased sensitivity to TMZ in the presence of melatonin. Melatonin treatment affects ABCG2 level via promoter methylation [42].
Procarbazine 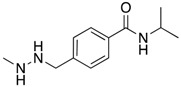	Alkylating agent GBM and MB	ABCB1	Primary tumor GBM cells treated with nimodipine, which blocks ABCB1, showed increased sensitivity to procarbazine in an MTT assay [43].
Lomustin/CCNU 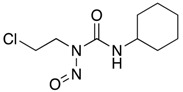	Alkylating agent GBM and MB	None reported in GBM/MB context	-
Carmustin/BCNU 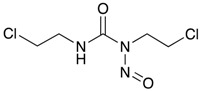	Alkylating agent GBM	ABCB1	GBM cancer stem cell line (U87CS), established by growing U87 GBM cell line in neuronal stem cell condition, showed over 8-fold increase in ABCB1 expression and exhibited greater resistance to BCNU [44].
Cyloposphamide 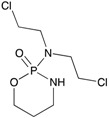	Alkylating agent MB	None reported in GBM/MB context	-
Carboplatin 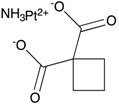	Platinum-based drugs. GBM, MB	ABCB1	GBM cancer stem cell line (U87CS), established by growing U87 GBM cell line in neuronal stem cell condition, showed over 8-fold increase in ABCB1 expression and exhibited greater resistance tow carboplatin [44].
Cisplatin 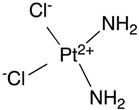	Platinum-based drugs. GBM, MB	None reported in GBM/MB context	-
Etoposide 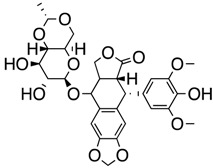	Topoisomerase inhibitor	ABCB1 ABCG2	Patients treated with cyclosporine A, which can inhibit both ABCB1 and ABCG2, had increased systemic exposure to etoposide [45] GBM cancer stem cell line (U87CS), established by growing U87 GBM cell line in neuronal stem cell condition, showed over 8-fold increase in ABCB1 expression and exhibited greater resistance to etoposide [44].
		ABCB1	ABCB1 expression is associated with high-risk MB in large patient cohorts.
		ABCB1	MB cell lines (DAOY, MED1, MED4, MED4R, MED5R, MED6) express high level of ABCB1. Treatment of these cells with etoposide in combination with ABCB1 inhibitors vardenafil or verapamil sensitizes cells to etoposide measured by clonogenic assay [38].
Topotecan 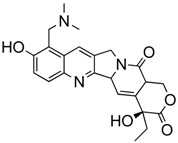	Topoisomerase inhibitor MB	ABCG2	Inhibition of ABCG2 in Group 3 MB tumorspheres by FTC increased sensitivity of topotecan measured by cell titer glow assay and annexin V staining for apoptotic cells [46]; ABCG2 inhibition with Ko143 increased cytotoxicity of topotecan in Group 3 MB preclinical animal model [46].
Irinotecan 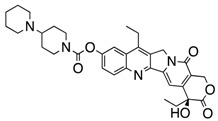	Topoisomerase inhibitor MB and GBM	ABCB1	CPT-11/SN-38 brain accumulation was found to be higher in *Mdr1a^+/−^* and *Mdr1^−^*^/*−*^ mice compared to *Mdr1a*^+/+^ mice [39].
		ABCB1	Sensitivity towards irinotecan increased in U118, U87, and SK72 GBM cell lines when ABCB1 was inhibited with pitavastatin as measured by alamar blue assay [47]. *ABCB1* mRNA level increased with irinotecan concentration [47].
Mitoxanthrone 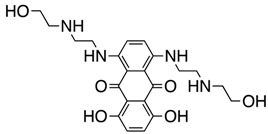	Topoisomerase inhibitor MB and GBM	ABCG2	SF395 human GBM cell line treated with mitoxanthrone resulted in ABCG2 being duplicated in mitoxanthrone-resistant clone [48].
		ABCG2	Group 3 tumorspheres treated with ABCG2 inhibitor, FTC, showed about 3-fold increase in sensitivity to mitoxanthrone [46].
Vinblastine 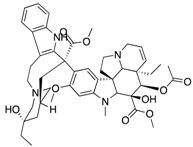	Vinca alkaloid/Anti-tubulin MB and GBM	ABCB1	*Mdr1a*^−/−^ mice showed 20-fold higher vinblastine level compared to *Mdr1a*^+/+^. The most striking difference in drug level was found in the brain [22].
Vincristine 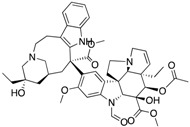	Vinca alkaloid/Anti-tubulin MB and GBM	ABCB1	Rats injected with vincristine showed elevated level of ABCB1 in the brain. Transport activity, monitored by radioactive tracer, 99 mTc-sestamibi, was subsequently found to increase 4-fold 24 h post vincristine treatment [49].
Paclitaxel (Taxol) 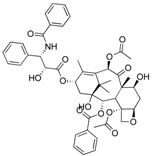	Anti-microtubule Glioma	ABCB1	Oral administration of ABCB1 inhibitor, zosuquidar, in mice, increased penetration of paclitaxel in the brain [50].
		ABCB1	Valspodar, an ABCB1 inhibitor, increased accumulation of paclitaxel in the brain of nude mice. Paclitaxel, in combination with valspodar, decreased tumor volume by 90% in nude mice bearing U118 MG glioblastoma [51].
Veliparib (ABT-888) 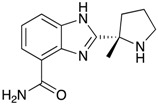	PARP1/2 inhibitor GBM	ABCB1 ABCG2	Tumors from spontaneous high grade glioma model *Pten; p16Ink4a*/*p19Arf; K-Rasv12* were isolated and implanted into nude mice which then received single dose of TMZ, ABT-888, or both with or without elacridar, a dual inhibitor of ABCB1 and ABCG2. Elacridar enhanced brain penetration of ABT-888. Co administration of elacridar enhances efficacy of TMZ and ABT-888 reduced glioblastoma tumor burden [52].
Lomeguatrib (*O*^6^Benzylguanine/O^6^BG) 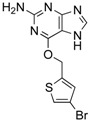	MGMT inhibitor GBM	ABCB1	O^6^BG can compete with Rhodamine 123 and pheophorbide A, substrates of ABCB1 and ABCG2 respectively, in uptake assays, indicating O^6^BG is a substrate of ABCB1 and ABCG2.
		ABCG2	Human glioblastoma GBP61 cells treated with verapamil and Ko143, which inhibit both ABCB1 and ABCG2 increased toxicity of *O*^6^BG and TMZ treatment [53].
Dasatinib 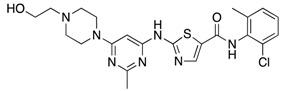	BCR-ABL/SRC kinase/Tyrosine kinase inhibitor (TKI) GBM	ABCB1 ABCG2	*Abcb1a/b^−/−^, Abcg2^−/−^, Abcb1a/b^−/−^; Abcg2^−/−^* mice received dasatinib by I.P and orally. Mice lacking both ABCB1 and ABCG2 showed higher accumulation in the brain than WT. Comparable level of dasatinib was observed in WT mice treated with ABCB1/ABCG2 dual inhibitor, elacridar and *Abcb1a/b^−/−^; Abcg2^−/−^* mice [54]
		ABCB1 ABCG2	mPDGFβ-induced de novo model of murine GBM induced in WT and *Abcb1a/b^−/−^; Abcg2^−/−^* (KO) mice were given 15 mg/kg dasatinib by oral gavage. Dasatinib level was double in both brain and tumor of KO mice compared to WT. KO mice treated with dasatinib also survived longer than WT [55]; In glioma cell lines from humans and murine models, treatment with elacridar (dual ABCB1 and ABCG2) inhibitor sensitizes cells to dasatinib [55]
Sunitinib 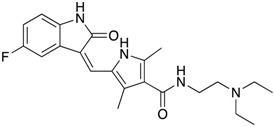	VEGFR, Flk1, PDGFR-α/β inhibitor Tyrosine kinase inhibitor (TKI) GBM	ABCB1 ABCG2	MDCK-II cells overexpressing human ABCB1 and ABCG2 showed transport of sunitinib. Addition of elacridar inhibited sunitinib transport. Brain accumulation of sunitinib was found to be significantly high in *Abcb1a/b^−/−^; Abcg2^−/−^* mice compared to WT. Elacridar oral administration in WT cells yields similar level of brain sunitinib concentration to *Abcb1a/b^−/−^; Abcg2^−/−^* mice [56]
		ABCB1 ABCG2	Sunitinib was administered to WT, *Abcb1a/b^−/−^, Abcg2^−/−^, Abcb1a/b^−/−^; Abcg2^−/−^* mice by IP infusion. Brain/plasma ratio was found highest in *Abcb1a/b^−/−^; Abcg2^−/−^* mice. Treatment with elacridar (dual ABCB1/ABCG2) inhibitor gave a brain/plasma ratio similar to *Abcb1a/b^−/−^; Abcg2^−/−^* mice [57]. Zosuquidar (ABCB1 specific inhibitor) only moderately increased sunitinib brain concentration while and Ko143 (ABCG2 inhibitor) had no effect [57].
Sorafenib 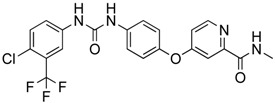	Tyrosine kinase inhibitor (TKI) GBM	ABCB1 ABCG2	WT, *Abcb1a/b^−/−^, Abcg2^−/−^, Abcb1a/b^−/−^; Abcg2^−/−^* mice were given sorafenib. Sorafenib accumulation did not increase in the brain of ABCB1-lacking mice, but increased moderately in *Abcg2^−/−^,* and most significantly in *Abcb1a/b^−/−^; Abcg2^−/−^* mice. Elacridar (dual ABCB1 and ABCG2 inhibitor) increased brain exposure to sorafenib similar to double knockout mice [58].

ABC (ATP-Binding Cassette), GBM (Glioblastoma), MB (Medulloblastoma), MTT (3-(4,5-dimethylthiazol-2-yl)-2,-5-diphenyltetrazolium bromide), BCNU (Bis-chlorethyl-nitrosourea), CCNU (chloroethyl-cyloexyl-nitrosourea), CPT (camptothecin), SN-38 (7-ethyl-10-hydroxycamptothecin, active metabolite of irinotecan), FTC (Fumitremorgin C), ABT (Abbott, now Abbvie), WT (wildtype), mPDGFβ (mouse platelet-derived growth factor beta).
